# Potential Role of Peptidylarginine Deiminase Enzymes and Protein Citrullination in Cancer Pathogenesis

**DOI:** 10.1155/2012/895343

**Published:** 2012-09-16

**Authors:** Sunish Mohanan, Brian D. Cherrington, Sachi Horibata, John L. McElwee, Paul R. Thompson, Scott A. Coonrod

**Affiliations:** ^1^Baker Institute for Animal Health and Department of Biomedical Sciences, Cornell University, Hungerford Hill Road, Ithaca, NY 14853-6401, USA; ^2^Department of Zoology and Physiology, University of Wyoming, Laramie, WY 82071, USA; ^3^Department of Chemistry, The Scripps Research Institute, FL 33458, USA

## Abstract

The peptidylarginine deiminases (PADs) are a family of posttranslational modification enzymes that catalyze the conversion of positively charged protein-bound arginine and methylarginine residues to the uncharged, nonstandard amino acid citrulline. This enzymatic activity is referred to as citrullination or, alternatively, deimination. Citrullination can significantly affect biochemical pathways by altering the structure and function of target proteins. Five mammalian PAD family members (PADs 1–4 and 6) have been described and show tissue-specific distribution. Recent reviews on PADs have focused on their role in autoimmune diseases. Here, we will discuss the potential role of PADs in tumor progression and tumor-associated inflammation. In the context of cancer, increasing clinical evidence suggests that PAD4 (and possibly PAD2) has important roles in tumor progression. The link between PADs and cancer is strengthened by recent findings showing that treatment of cell lines and mice with PAD inhibitors significantly suppresses tumor growth and, interestingly, inflammatory symptoms. At the molecular level, transcription factors, coregulators, and histones are functional targets for citrullination by PADs, and citrullination of these targets can affect gene expression in multiple tumor cell lines. Next generation isozyme-specific PAD inhibitors may have therapeutic potential to regulate both the inflammatory tumor microenvironment and tumor cell growth.

## 1. Introduction

**Figure 1:**
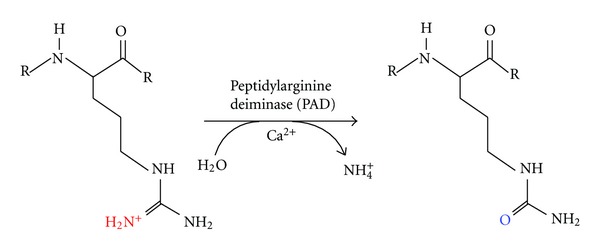
Peptidylarginine deiminase (PAD) enzymes catalyze the conversion of protein arginine residues to citrulline.

PAD-mediated citrullination can alter the tertiary structure of target substrates and/or alter protein-protein interactions; thus affecting various cellular processes [1, 2] (Figure 1). Recently, protein citrullination has garnered increased attention due to its role in the pathogenesis of various inflammatory conditions such as rheumatoid arthritis (RA), multiple sclerosis, psoriasis, chronic obstructive pulmonary disease (COPD), neurodegenerative diseases and, also, due to its emerging role in various human and animal cancers [3–7]. In this paper, we will first briefly discuss the tissue-specificity and hormonal regulation of the five PAD isoforms and then focus on the potential role of this enzyme family in carcinogenesis, tumor progression, and inflammation.

## 2. Tissue Expression Patterns and Substrate Specificity of PAD Family Members

PADs are Ca^2+^-dependent enzymes and there are five different isozymes in mammals, namely, PAD1, 2, 3, 4, and 6 [8, 9]. The *PAD* genes likely arose by duplication of the ancestral homologue, *PAD2*, and are localized to a well-organized gene cluster at 1p36.13 in humans. Interestingly, this locus is also predicted to contain a novel, yet to be defined, tumor suppressor protein [10]. PAD enzymes are highly homologous, with ~50–60 percent sequence identity at the amino acid level. Although there is some overlap with respect to target proteins, each family member also appears to target a unique set of cellular proteins as well [11, 12]. Additionally, as described below, each family member also exhibits a tissue-selective distribution pattern. We note here that, in this paper, we will be using the more commonly utilized gene synonym, *PAD*, while the approved HUGO Gene Nomenclature Committee gene names for the family members are actually *PADI1, PADI2, PADI3, PADI4,* and *PADI6*.

PAD1 appears to primarily be expressed in the epidermis and uterus. In the epidermis, PAD1 citrullinated cytokeratin and this modification is important for modulating the cornification of the epidermis and maintaining the barrier function of superficial keratinized epidermal cell layers. The loss of charge following citrullination of cytokeratin causes disassembly of the cytokeratin-filaggrin complex and proteolytic degradation of these targets.

PAD2 is expressed in multiple organs, including the brain, female reproductive tissues, skeletal muscle, and cells of the hematopoietic lineage. In the brain, a major target for PAD2 is myelin basic protein (MBP), a constituent of the myelin sheath [13, 14]. Citrullination of MBP by PAD2 likely plays a key role in the pathogenesis of neurodegenerative disease [15]. PAD2 can also citrullinate the intermediate filament vimentin in macrophages [16], leading to cytoskeletal disintegration and eventually apoptosis. In female reproductive tissues, PAD2 levels appear to be regulated by hormones, predominantly estrogen, and also possibly by epidermal growth factor [17]. PAD2 has also been found to be expressed in human mammary gland epithelial cells, with a fraction of PAD2 in these cells localizing to the nucleus and binding directly to chromatin [18]. In canine mammary tissue, histone citrullination levels closely correlate with the expression of PAD2 across the estrous cycle, suggesting that PAD2 may target histones for citrullination in this tissue [17]. In support of this prediction, a recent study has found that PAD2-catalyzed citrullination of histone H3 arginine 26 can regulate estrogen receptor *α* target gene activity [19].

PAD3 expression is highly restricted to the hair follicle and epithelium and a major target for PAD3 is trichohyaline. Additionally PAD3 can also citrullinate filaggrin leading to altered epidermal homeostasis and loss of barrier function [20].

PAD4 is expressed in hematopoietic progenitor cells, immune cells such as granulocytes, monocytes and macrophages, natural killer cells, and carcinoma cells originating from lung, esophagus, breast, and ovary [5, 21]. PAD4 is often localized to the nucleus and is the only PAD family member with a canonical nuclear localization sequence [22]. Antithrombin has been found to be an extracellular PAD4 substrate [23] and citrullination of this target suppresses the ability of antithrombin to inhibit thrombin [24]. Increased thrombin activity is considered to be a hallmark of cancer by promoting angiogenesis, increased tumor growth, and distant metastasis. Interestingly, citrullinated antithrombin levels are elevated in serum samples from patients with malignant cancers, thus raising the possibility that PADs may affect tumor progression via citrullination of antithrombin [25].

**Figure 2:**
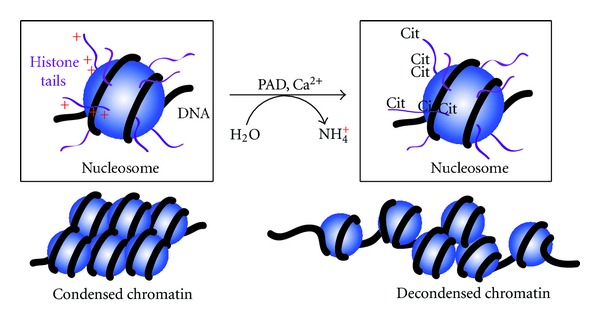
PAD-mediated histone tail citrullination leads to chromatin decondensation.

PAD4 also appears to function as a transcriptional coregulator for a range of factors such as p53, ELK1, p300, p21, CIP1, nucleophosmin, and ING4 [26–30]. While the mechanism by which PAD4 regulates target gene activity is not entirely clear, Edman degradation and analysis using site-specific anticitrullinated histone antibodies has found that PAD4 can target the N-terminal tails of histones H2A, H3, and H4 for citrullination. More specifically, PAD4 has been found to directly citrullinate histone H4 and H2A at arginine 3, and histone H3 at arginines' 2, 8, 17, and 26. Histone tail citrullination has been found to promote chromatin decondensation *in vitro* and *in vivo* [31, 32]. Thus, it seems likely that the gene regulatory role of PAD4 is mediated by its initial recruitment to target promoters by the relevant transcription factor, followed by subsequent deimination of specific residues in the N-terminal histone tails, leading to local changes in chromatin architecture and modulation of target gene expression (Figure 2). PAD4 is both a corepressor and coactivator of gene transcription and also appears to contribute to epigenetic cross-talk [33] during DNA damage by acting in concert with histone deacetylase 2 (HDAC2) to regulate p53 target gene activity [34]. Following DNA damage, PAD4 and HDAC2 separate from the p53-target gene promoters such as p21, GADD45, and PUMA, resulting in an increased incidence of histone Lys acetylation and Arg methylation at these sites.

*PAD6* is a maternal effect gene that is specifically expressed in oocytes and preimplantation embryos and is essential for embryonic development beyond the 2-cell stage [35]. To date, there has been little evidence that this ovarian PAD isozyme is involved in cancer. Interestingly, however, a genome-wide SNP association in Icelanders showed a significant correlation between cutaneous-basal cell carcinoma risk and mutations in the PAD4/PAD6 locus at 1p36 [36]. The importance of this PAD family member in developmental biology is reviewed elsewhere [37, 38] and will not be discussed further in this paper.

## 3. Hormonal Regulation of PADs

Early PAD biological studies focused mainly on the expression patterns of the different family members in reproductive tissues and also on their catalytic activity at these sites. Outcomes from these studies showed that anterior pituitary-localized rat lactotroph cells express PAD2 in a sexually dimorphic fashion, with expression confined to the female. Additional studies showed that PADs 1, 2, and 4 are present in uterine tissue and that protein citrullination is extensive in the uterine epithelium in an estrus cycle-dependent manner [39, 40]. More recently, studies have found that mouse, dog, and human mammary glands express both PAD2 and 4 in luminal epithelial cells [17].

### 3.1. Pituitary Gland and Uterus

In the female rat pituitary, PAD enzymatic activity is highest during proestrus and estrus when serum estrogen levels are at their peak [39, 41]. Ovariectomy of rats suppressed PAD activity in pituitary lysates, but activity could be restored by injection of exogenous estrogen. Furthermore, treatment of the pituitary-derived MtT/S cell line with estrogen results in a dose-dependent increase in PAD expression and activity [42]. Together, these observations suggest that estrogen directly influences PAD expression and activity in the pituitary. Given that estrogen primes lactotrophs for prolactin biosynthesis, PAD enzymatic activity may play an important role in this normal physiological process and may also promote pituitary neoplastic growth.

Our examination of Massively Parallel Signature Sequencing (MPSS) transcriptome data (GEO Profiles GDS868) indicates that PADs 1, 2, and 4 expression levels are highest in the mouse uterus as compared to the 50 other tissues examined [43]. Furthermore, analysis of cDNA microarray data from Hewitt et al. shows that PAD1, 2, and 4 mRNA appear to be estrogen regulated [44]. In the uterus, the distribution of these isozymes appears to be primarily limited to glandular and luminal epithelial cells and also displays an estrous cycle-dependent regulation pattern, with the highest expression of these family members occurring during estrus [45, 46]. Similar to the pituitary, PAD2 and PAD4 expression and activity in the uterus is lost following ovariectomy, but can be restored by injection of exogenous estrogen, indicative of estrogen regulation [45, 46]. Given the known mitogenic properties of estrogen in female cancers, it is possible that estrogen-induced upregulation of PAD expression and activity in the uterus may promote neoplastic growth in this tissue. However, a potential role for PADs in pituitary and uterine tumor progression has yet to be investigated.

### 3.2. Mammary Gland

Protein and mRNA expression studies in canine mammary glands collected at various stages of the estrous cycle show that PAD2 expression initiates during estrus, with mRNA and protein levels peaking during diestrus. In the mouse mammary gland PAD2 and 4 expression is highest in luminal epithelial cell populations during the estrus [17, 46]. This species-specific difference in expression levels may reflect differences in estrous cycle stage lengths and hormone levels between species. Ovariectomy in mice also results in loss of PAD2 and 4 expression in the mammary gland, thus further corroborating the role of estrogen in PAD expression in female reproductive tissues. Molecular studies show that treatment of MCF-7 cells with estrogen rapidly induced the upregulation of PAD4 and the involvement of estrogen in PAD4 expression in mammary epithelial cells appears to be mediated by two upstream estrogen response elements (designated ERE −125 and −126) which are bound by ER*α* following estrogen treatment. Furthermore, this study showed that the ER*α*-dependent increase in PAD4 expression can also be mediated by estrogen signaling to the PAD4 proximal promoter via cross-talk with the AP-1, Sp-1, and NF-Y transcription factors [47].

## 4. PAD4-Mediated Histone Tail Citrullination: An Emerging Role for PADs in Gene Regulation and Cancer

PAD4 has also been found to regulate estrogen receptor target gene activity following estrogen stimulation via histone tail citrullination [48]. Given the role of estrogen as a mitogen in cancer cells, this observation provides a clear potential link between PAD activity and cancer growth. In addition to estrogen, another mitogen, EGF, has been shown to utilize PAD4 as a cofactor to activate target gene activity. Zhang et al. documented that treatment of MCF-7 cells with EGF leads to PAD4-mediated citrullination of the ELK1 oncogene. This citrullination event then facilitated subsequent phosphorylation of ELK1 by ERK1/2, which, in turn, promoted histone acetylation and subsequent activation of a range of targets including the immediate early gene, c-fos [30].

**Figure 3:**
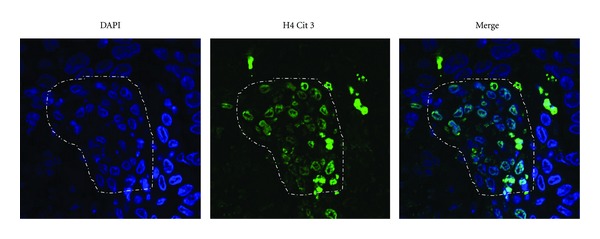
H4cit3 immunostaining of DCIS xenograft sections. Dotted line demarcates the area adjacent to the central necrotic core of the comedo-DCIS lesion. (Magnification 400x).

PAD4 has also been found to interact with the major tumor suppressor, p53, and affect the expression of p53 target genes such as *p21*, *OKL38*, *CIP1,* and *WAF1* [26–30]. Interestingly, a recent study also found that citrullination levels at histone H4 arginine 3 (H4R3) are inversely correlated with p53 protein expression and with tumor size in nonsmall cell lung cancer tissues [32]. The authors also demonstrated that the p53-PAD4 pathway leads to citrullination of H4R3 and Lamin C in response to DNA damage and nuclear fragmentation. They also found that PAD4-mediated H4R3 citrullination appears to lead to localized chromatin decondensation around sites of DNA damage, thus facilitating p53-mediated cell death. *In vivo* studies then demonstrated that even though PAD4-null mice were grossly normal with regard to organ morphologies, they appeared resistant to apoptotic stimuli and also showed a consistent reduction in cleaved caspase-3 expression. The authors conclude that the histone H4 citrulline 3 (H4Cit3) modification may form a novel “apoptotic code” which could potentially be used to detect a range of damaged cells, including tumor cells, following treatment of patients with cancer therapies. It is interesting to note, however, that the PAD inhibitor Cl-amidine increases p53 levels both in cell culture and in inflammatory cells isolated from mice treated with this compound [29, 49]. Reconciling these two disparate observations requires further study. In support of a role for the H4Cit3 modification in marking apoptotic tumor cells, recent immunofluorescence studies in our lab found that citrullination at H4R3 was very robust within the nucleus of epithelial cells undergoing morphological changes associated with various stages of apoptosis in comedo-DCIS xenograft sections (Figure 3). These xenografts were generated from MCF10DCIS cell line which belongs to the group of MCF10AT tumor progression series of cell lines [50, 51].

From a more clinical perspective, recent immunohistochemical and western blot studies have found that PAD4 appears to be overexpressed in several types of invasive carcinomas [5, 52, 53]. Outcomes from these studies found that PAD4 expression and, frequently activity, was elevated in neoplastic cells from breast carcinomas, lung adenocarcinomas, hepatocellular carcinomas, esophageal carcinomas with squamous differentiation, colorectal adenocarcinomas, renal carcinomas, ovarian adenocarcinomas, uterine carcinomas, uterine adenocarcinomas, and bladder carcinomas [52]. However, PAD4 expression was absent or minimal in the following tumors: benign gastric and uterine leiomyomas, hyperplastic conditions of endometrium, cervical polyps, teratomas, hydatidiform moles, hemangiomas, lymphatic proliferative conditions, schwannomas, and neurofibromas [5]. Consistent with the PAD4 protein expression pattern in carcinomas, the same patients also had elevated serum PAD4 activity and citrullinated antithrombin levels. Taken together, these observations support the potential use of this enzyme as a clinical prognostic biomarker.

## 5. PAD2 and Cancer Pathogenesis

**Figure 4:**
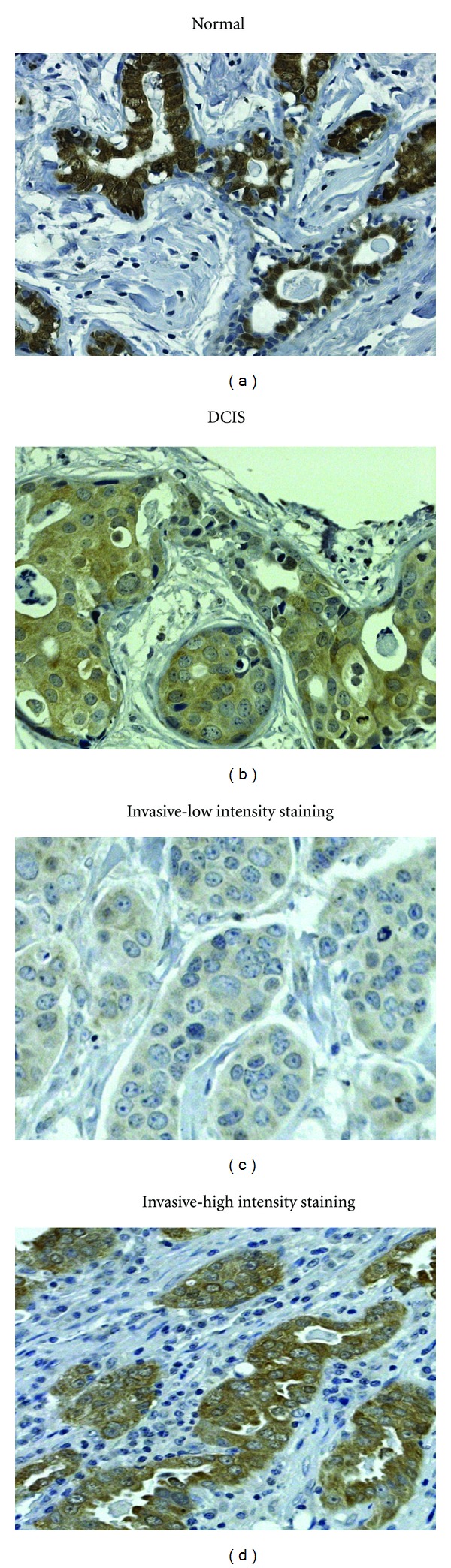
PAD2 IHC staining of the normal human mammary gland, a DCIS lesion, and invasive carcinomas. Nuclear staining intensity is reduced in most invasive tumors while a subset of these tumors retains strong PAD2 staining. (Magnification 200x).

**Figure 5:**
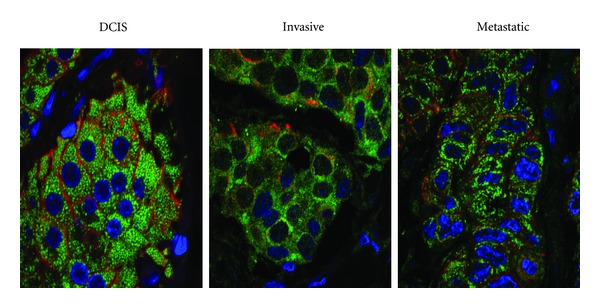
PAD2 (green) is expressed in luminal epithelial cells of preinvasive, invasive, and metastatic human mammary tumors. Cytokeratin (red) staining differentiates luminal epithelial type from myoepithelial cells. (Magnification 400x).

Given the volume of recent literature linking PAD4 with gene regulation in cancer cells, a role for this family member in tumor progression seems likely. Comparative studies evaluating PAD2 expression in mammary carcinomas from humans, dogs, and cats show that the nuclear localization of PAD2 may also prove to be associated with tumor progression. Normal human, canine, and feline mammary tissue shows strong nuclear and cytoplasmic PAD2 staining, but as tumors progress there is a general reduction in the nuclear localization of PAD2 (Figures 4 and 5) [54]. In the mammary gland, PAD2 expression is specific to cytokeratin positive luminal type epithelial cells (Figure 5). Thus, the loss of nuclear PAD2 may result in alterations in gene expression that lead to neoplastic transformation. It is interesting to note that a subset of invasive breast carcinomas tend to retain strong cytoplasmic and nuclear expression of PAD2. Further characterization of this subclass of tumors with regard to function of PAD2 is warranted as isozyme specific PAD inhibitors may be of use in combinatorial therapies to treat such tumor types.

Given these new findings, and PAD4's documented role in cancer biology, it will be interesting to determine whether PAD2 and PAD4 may function in a synergistic manner to promote tumor progression. The observation that both PAD2 and PAD4 expression appears to be regulated at some level by estrogen, suggests that these two PADs may work together to mediate the estrogen response. The observations that PAD2 expression in mammary epithelial cells is induced by EGF [17], and that PAD4 regulates EGF-induced ELK1 target gene activation, suggests that PAD2 and PAD4 may also cooperate to mediate EGFR signaling [30].

## 6. PAD Inhibitors Block Cancer Progression

In further support of a role for PADs in tumor growth, several recent reports have also shown that treatment of cancer cell lines with PAD inhibitors decreases cancer cell viability without affecting the growth of normal cells [55]. Cl-amidine [56], and the related PAD4 inhibitor, F-amidine [57], display low micromolar cytotoxicity towards various tumor cell lines such as U2OS cells, HL-60, HT-29, and MCF-7 [29, 55, 58]. These compounds also can induce the differentiation of HL-60 cells, a leukemic cell line, making these cells more susceptible to drug treatments [55]. Cl-amidine can also act synergistically with the anticancer drug doxorubicin, thus enhancing the efficiency of cell death following a simultaneous treatment with these two compounds. In tumor, cell lines such as MCF-7 cells, Cl-amidine also regulates the expression of the tumor suppressor protein OKL38 in a p53-dependent manner by decreasing histone citrullination at the OKL38 promoter [28, 29].

**Figure 6:**
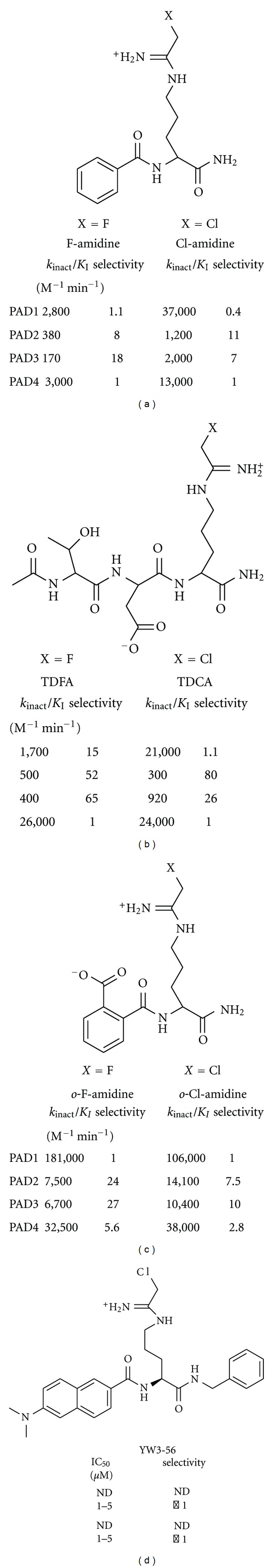
PAD inhibitors and their selectivity (TDFA-Threonine-aspartate-F-amidine; TDCA-Threonine-aspartate-Cl-amidine).

A recent study, using a Cl-amidine derivative with increased cell permeability, YW3-56 (Figure 6), found that this drug significantly suppressed cancer cell growth and also reduced tumor size in mouse xenograft models of sarcoma [59]. Furthermore, this compound affected the expression of genes related to cell proliferation and cell death and was also found to regulate macro-autophagy in cancer cells. Mechanistically, the authors discovered that the drug likely targeted factors within the mTORC1 pathway for inhibition. These studies, as well as the demonstration that PAD inhibitors are well tolerated in multiple different mouse disease models [49, 60], underscores the potential of PAD inhibitors as novel epigenetic anticancer drugs. Given that Cl-amidine, F-amidine, and YW3-56 display limited selectivity (Figure 6), it is unclear whether the inhibition of one or more PADs is required for the *in vivo *effects of these compounds. The development of new, more selective compounds, such as the PAD4 selective inhibitor TDFA and the PAD1 selective inhibitor o-F-amidine (Figure 6), will undoubtedly prove to be useful for sorting this issue out.

## 7. PAD-Mediated Citrullination: Linking Inflammation with Cancer Progression?

### 7.1. Role of Inflammation in Cancer Progression

Chronic inflammation is involved in the progression and recurrence of many types of cancer, including breast cancer. Epidemiological studies have documented that high levels of circulating acute phase inflammation-associated proteins at 3 years after-treatment are associated with an elevated risk for subsequent tumor recurrence and mortality in women [61]. Several studies have also demonstrated direct links between circulating inflammatory markers and progression to metastatic breast cancer [62–66]. Additionally, proinflammatory cytokines are well known for promoting tumor growth and facilitating metastasis by altering the tumor cell phenotype and by regulating stromal cells (endothelial cells, tumor-associated macrophages, and fibroblasts) within the tumor microenvironment. Furthermore, infiltrating immune cells within the tumor itself can promote tumorigenesis [67]. These findings suggest that the mechanisms by which inflammation in the tumor microenvironment drives metastasis is both intimately linked and fundamentally different to the primary mechanisms driving carcinogenesis [68].

### 7.2. Role of PADs in Inflammation

As noted earlier, numerous studies have documented increased protein citrullination within inflamed tissues from patients with autoimmune diseases such as rheumatoid arthritis and colitis [3, 4, 69]. More recently, these inflammatory symptoms have been shown to be suppressed by the PAD inhibitor, Cl-amidine, in mouse models of colitis and RA [49]. Another emerging link between PADs and inflammation is the newly defined role for PAD4 in catalyzing histone hypercitrullination during Neutrophil Extracellular Trap (NET) formation in inflamed tissues. A number of recent reports have shown that, following activation, peripheral blood neutrophils form a highly decondensed chromatin structure that both captures and kills invading pathogens [70–72]. Mechanistically, PAD4 was found to catalyze this dramatic chromatin decondensation event via histone tail hypercitrullination [73]. Analysis of this process at the ultrastructural level by electron microscopy showed that, following PAD4 activation in HL60 granulocytes, these cells show a dramatic and rapid conversion of multilobular heterochromatic nuclei to a more round euchromatic nuclear architecture, suggesting a direct role for PADs in heterochromatic-euchromatic interchange [31]. These new findings indicate that PAD4 can mediate chromatin structure change both at the local and genome-wide level.

Citrullination of vimentin is correlated with the proliferation of fibroblast-like synoviocytes (following isolation from patients with rheumatoid arthritis) and also stimulates TNF-*α* and IL-1 production in these cells [74]. Given the links between vimentin citrullination, inflammation, and cell proliferation, and given how important cytoskeletal integrity is for cell motility, we hypothesize that citrullination of vimentin by PAD enzymes may also affect tumor cell migration and promote an inflammatory microenvironment. Additionally, given that PAD2 can regulate cytokine signaling in macrophages via citrullination of IKK*γ* (thus suppressing NF-kB activity) [75], we predict that PAD-mediated regulation of macrophage activity could also potentially affect cross-talk between tumor-associated macrophages and cancer cells.

While PAD activity has primarily been found to regulate autoimmune-mediated inflammatory events, recent studies suggest that PAD-mediated citrullination is also elevated in a variety of inflammatory states which lack a strong autoimmune component, such as COPD and myositis [76–78]. Perhaps the best demonstration that PAD-mediated citrullination can facilitate nonautoimmune or microbial-induced inflammatory events is the recent finding that PAD activity is strongly upregulated in inflamed tissue following a sterile skin punch biopsy procedure in mice [79]. Thus, it can be inferred that PAD-mediated citrullination plays a critical and fundamental role in inflammatory events induced by a range of pathologies, both infectious and noninfectious.

### 7.3. Role of PADs in Chemokine Signaling

PAD2 and PAD4 are highly expressed in peripheral blood mononuclear cells such as NK cells, T cells, B cells, and monocytes [16] and, thus, are likely to be the main PAD “players” in chemokine signaling. Chemokines are important for the proper recruitment of leukocytes to the site of inflammation. The chemokine-receptor system can be dramatically modified in neoplasms, especially at the invasive edges, and can act as a proangiogenic and a prodesmoplastic mediator. Several chemokines, including CXCL1, 2, and 8 have been found to exert effects on tumor cell growth. The CXC group of chemokines, with the tripeptide (Glu-Leu-Arg/ELR) at the amino-terminus of the CXC motif (ELR+), is proangiogenic and stimulates cell migration and proliferation. The capacity of chemokines to activate or repress biological pathways depends, in part, upon posttranslational modifications such as glycosylation and on proteolytic processing of the chemokine's N- or C-terminus [80]. Importantly, PADs have recently been found to citrullinate CXCL5, CXCL8, CCL17, and CCL26 [81], thus directly modulating the inflammatory milieu. Furthermore, chemokine citrullination does not appear to be a rare event *in vivo,* as, for example, CXCL8 was found to be citrullinated at arginine 5 in 14% of all blood leukocyte derived CXCL8 [81]. While the role of citrullinated chemokines in the inflammatory process is currently coming to light, their effect in cancer progression has yet to be investigated. However, given the strong links between inflammation and cancer progression, these observations support the hypothesis that PAD activity may play an important role in regulating the inflammatory milieu of the cancer microenvironment.

## 8. Conclusions

**Figure 7:**
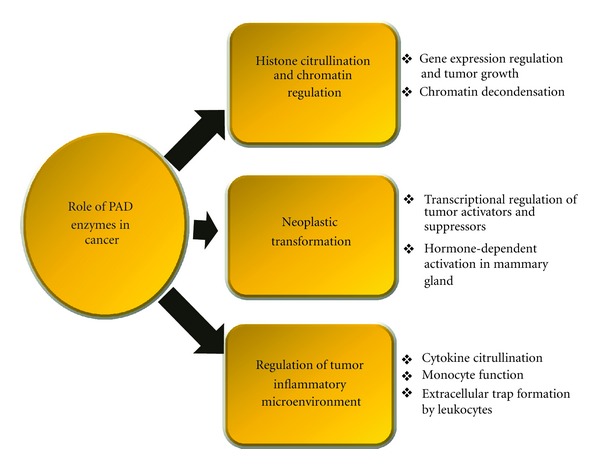
Potential role of PAD enzymes in cancer pathogenesis.

Protein citrullination is emerging as a critical posttranslational modification in developmental biology, inflammation, and cancer pathogenesis. With respect to cancer, PAD enzymes are now being identified as important potential players in tumor progression that both regulate transcriptional activity and modulate the inflammatory microenvironment via cytokine citrullination. The major likely roles of PADs in cancer pathogenesis are summarized in Figure 7. Given these emerging links between PADs and cancer biology, a better understanding of the upstream mechanisms that induce PAD expression, and the downstream mechanisms by which PADs regulate gene expression and inflammatory events will likely advance our understanding of tumor biology. Furthermore, the upregulation of specific PAD isozymes and activity at critical points of tumor progression raises the possibility that these enzymes, and their resulting posttranslational modifications, can function as novel cancer biomarkers. Lastly, the observations that (1) PAD inhibitors reduce inflammatory symptoms in mouse models of disease, (2) the link between PADs, inflammation, and cancer is currently unfolding, and (3) next generation isozyme-specific PAD inhibitors are currently being developed, raise the possibility that the use of PAD inhibitors in preclinical and clinical cancer therapies may soon be realized.
